# YAP Enhances Autophagic Flux to Promote Breast Cancer Cell Survival in Response to Nutrient Deprivation

**DOI:** 10.1371/journal.pone.0120790

**Published:** 2015-03-26

**Authors:** Qinghe Song, Beibei Mao, Jinbo Cheng, Yuhao Gao, Ke Jiang, Jun Chen, Zengqiang Yuan, Songshu Meng

**Affiliations:** 1 State Key Laboratory of Brain and Cognitive Sciences, Institute of Biophysics, Chinese Academy of Sciences, Beijing 100101, China; 2 College of Life Sciences, University of Chinese Academy of Sciences, Beijing 100049, China; 3 Institute of Cancer Stem Cell, Dalian Medical University Cancer Center, Dalian, Liaoning, China; 4 Center of Growth, Metabolism and Aging, College of Life Sciences, Sichuan University, Chengdu, China; University of Manitoba, CANADA

## Abstract

The Yes-associated protein (YAP), a transcriptional coactivator inactivated by the Hippo tumor suppressor pathway, functions as an oncoprotein in a variety of cancers. However, its contribution to breast cancer remains controversial. This study investigated the role of YAP in breast cancer cells under nutrient deprivation (ND). Here, we show that YAP knockdown sensitized MCF7 breast cancer cells to nutrient deprivation-induced apoptosis. Furthermore, in response to ND, YAP increased the autolysosome degradation, thereby enhancing the cellular autophagic flux in breast cancer cells. Of note, autophagy is crucial for YAP to protect MCF7 cells from apoptosis under ND conditions. In addition, the TEA domain (TEAD) family of growth-promoting transcription factors was indispensable for YAP-mediated regulation of autophagy. Collectively, our data reveal a role for YAP in promoting breast cancer cell survival upon ND stress and uncover an unappreciated function of YAP/TEAD in the regulation of autophagy.

## Introduction

The Hippo/MST pathway plays a key role in tissue homeostasis and organ size control [1,2]. A major downstream target of the Hippo/MST pathway is the mammalian transcriptional coactivator Yes-associated protein (YAP). YAP shuttles between the cytoplasm and nucleus, where it activates the TEA domain (TEAD) family of growth-promoting transcription factors to regulate gene expression [3]. The human chromosome 11q22 amplicon, which encompasses the *YAP* gene, is frequently amplified in multiple types of human cancers [4–6]. Furthermore, increased expression and/or nuclear accumulation of YAP were observed in a variety of human cancers [7–9], indicating a role for YAP as a candidate oncogene in tumorigenesis.

In breast cancer, however, the role of YAP in cancer development remains controversial. Loss of heterozygosity (LOH) of the *YAP* gene locus (located at 11q22.2) was frequently detected in sporadic breast cancer [10–14], suggesting that YAP may function as a tumor suppressor. In addition, reduced expression of YAP in invasive carcinoma is associated with estrogen receptor α (ERα) and progesterone receptor (PR) negativity in invasive breast carcinomas [15]. Loss of YAP expression contributes to the suppression of anoikis, the increased migration and invasiveness and the enhanced tumor growth in nude mice [16]. These findings support a role of YAP as tumor suppressor gene in breast cancer. However, contrasting observations suggest an oncogenic role for YAP in breast cancer [17]. Over-expression of YAP in human non-transformed mammary epithelial MCF-10A cells leads to phenotypic alterations that are hallmarks of tumorigenic transformation [6]. In addition, a very recent report showed that loss of YAP suppresses oncogene-induced tumor growth in mouse mammary glands [9]. Collectively, the role of YAP in breast cancer is complex and needs to be further investigated.

Macroautophagy (hereafter referred to as autophagy) is an evolutionarily conserved lysosome-dependent cellular catabolic degradation pathway. The hallmark of autophagy is the formation of double- or multi-membrane vesicles in the cytosol, called autophagosomes, which encapsulate bulk cytoplasm and cytoplasmic organelles [18]. The outer membrane of the autophagosome then fuses with endosomes or lysosomes to form autolysosomes that degrade their content. The degradation products can be used as sources of energy during periods of metabolic stress (e.g., starvation), thereby promoting cell survival [19,20]. In a tumor microenvironment, autophagy can promote cancer cell survival in response to harmful stress [21–23]. Autophagy delays apoptotic death in noninvasive breast cancer cells following DNA damage [24]. On the other hand, progressive autophagy can also induce cell death [25]. Therefore, there is likely a balance between oncogenic and tumor suppressive autophagy during tumorigenesis.

Recently, Maejima et al. showed that mammalian STE20-like kinase-1 (MST-1) subverts autophagy and promotes apoptosis in the heart [26], suggesting a role for the MST/YAP pathway in integrating autophagy and apoptosis during cellular stress. This finding motivated us to analyze whether YAP could modulate autophagy in a cancer setting. In this work, we have found that autophagy is critical for YAP to protect MCF7 breast cancer cells from apoptosis under nutrient deprivation conditions. Importantly, YAP modulates autophagic flux by enhancing autolysosome degradation. Therefore, our study suggests a role for YAP in regulating autophagy and promoting breast cancer cell survival under nutrient deprivation conditions.

## Materials and Methods

### Cell culture and reagents

Human breast cancer MCF7 and MDA-MB-231 cells were purchased from American Type Culture Collection (ATCC) and maintained in Dulbecco’s modified Eagle’s medium (DMEM) (Invitrogen) with 10% fetal bovine serum (FBS, Invitrogen) and penicillin/streptomycin in a humidified incubator under 95% air and 5% CO_2_ at 37°C. For nutrient deprivation (ND), cells were incubated in Earle’s Balanced Salt Solution (EBSS, without glucose) after 2 times of washing with EBSS. All other cell culture materials were obtained from Thermo and all chemicals were obtained from Sigma-Aldrich.

Concentrations of chemicals used to treat cells are as follows: Chloroquine (CQ, 25μM), Bafilomycin A1 (BafA1, 10nM), Rapamycin (Rapa, 20μM), 3-Methyladenine (3-MA, 5mM), NH_4_Cl (20mM).

### Lentiviral shRNA cloning, production, and infection

All short hairpin RNAs (shRNA) were cloned into plko.1-vector. TEADs shRNAs were designed in a region identical in TEAD1, 3, and 4.The targeted sequences are as follows: shYAP, 3’-GACATCTTCTGGTCAGAGA-5’; shTEADs, ATGATCAACTT CATCCACAAG; scrambled control RAN interference sequence (shCtrl), 3’-GACATTTGTAACGGGATTC-5’.The lentivirus production and infection were performed as described previously [27]. Resulting control and YAP or TEADs knockdown cell lines were called shCtrl, shYAP and shTEADs respectively.

### Retrovirus infection

To generate wild-type (WT) or mutant YAP-expressing stable cells, retrovirus infection was performed as previously reported [28]. The pQCXIH empty vector, pQCXIH-YAP WT and S94A constructs are gifts from Zhao Bin (Zhejiang University, China). The control and WT YAP-overexpressing stable cell lines were called Ctrl and YAP, respectively. The YAP rescue mutant was obtained as described previously [27]. MCF7-shYAP cells were separately infected with retrovirus expressing YAP-WT and YAP-S94A to generate the stable cell lines WT Res and S94A Res.

### Stable cell lines

MCF7-shCtrl and shYAP cells were stably transfected with plasmid expressing tandem fluorescent mRFP-GFP-tagged LC3 (tfLC3) (a gift from Dr. T. Yoshimori from Osaka University, Osaka, Japan) [29]. The resulting cell lines were called MCF7-shCtrl-tfLC3 and MCF7-shYAP-tfLC3. Cells with high-fluorescence-intensity puncta in the MCF7-shYAP-tfLC3 cell line were selected by flow cytometry. The resulting high-fluorescence-intensity-puncta cell line was named tfLC3-shYAP, and subsequently stably infected with retrovirus expressing YAP-WT or YAP-S94A to generate the tfLC3-WTr and tfLC3-S94Ar cell lines.

### Transfections

siRNA transfections were performed with Lipofectamine 2000 (Life Technologies) according to the manufacturer's instructions. Sequences of siRNA are as follows: LC3 1#, 3’-uaauuagaaggcgcuuaca-5’; LC3 2#, 3’-uagaacgauacaaggguga-5’.

### MTT assay

The cell viability was determined by MTT assay. Cells were seeded in 96-well flat plate for 24 h. After designated treatments, the MTT assay was performed as previously described [30]. Quantitative analysis of relative cell viability was derived from triplicates. The experiments were repeated three times.

### Immunofluorescence

Immunofluorescence were conducted as described previously [31].

### Hoechst staining

Apoptotic nuclear changes were examined using Hoechst 33258 (Sigma). The cells were fixed with 4% paraformaldehyde, stained with Hoechst 33258 for 10 min. All the nuclear morphology images were obtained using a fluorescence microscope (Carl Zeiss).

### Cell apoptosis assay

Apoptosis was determined by flow cytometric analysis of membrane redistribution of phosphatidylserine using an Annexin V-FITC and propidium iodide (PI) double staining technique. The experiment was performed as described previously [30]. Apoptotic cells were expressed as the percentage of both early stage (PI negative and Annexin V positive) and late stage (PI positive and Annexin V positive) populations. Surviving cells were the PI negative and Annexin V negative population. Quantitative analysis of percentage of apoptotic cells was derived from triplicates. The experiments were repeated three times.

### Western blotting

Western blot analyses were conducted as described previously [32]. Densitometry analysis of the Western blotting bands was performed using ImageJ software. The quantified results were calculated from three independent experiments. The primary antibodies were used as follows: YAP (NOVUS, NB110-58308), TEAD4 (Sigma, WH0007004M1), microtubule-associated protein1 light chain 3 (LC3) (Cell Signaling, #2775), p62 (MBL, pm045), S6 ribosomal protein (Cell Signaling, #2217), and phospho-S6 ribosomal protein (ser235/236) (Cell Signaling, #2211).

### Confocal microscopy

Cells stably expressing tandem fluorescent mRFP-GFP-tagged LC3 (tfLC3) were seeded in glass bottom cell culture dishes (NEST, 801002) and cultured for 24 h. After treatments, fluorescent images were directly taken from live cells using an inverted confocal microscope (Leica). For quantification of autophagic cells, fluorescent puncta were determined from triplicates by counting more than 30 cells per field.

### High content cells assay

MCF7-tfLC3-shYAP, tfLC3-WTr and tfLC3-S94Ar cells were seeded in 24-well plates and cultured for 24 h. After the designated treatments, average red and green fluorescent intensity per live cell in each well was directly measured by a high content cell assay microscope system (Thermo). The quantified results were given as the ratios of GFP/RFP fluorescent intensity from triplicates.

### Acid phosphatase activity assay

MCF7-shCtrl, shYAP and YAP Res cells were seeded in 6-well plates and cultured for 24 h. The lysates were assayed by the Acid Phosphatase Assay Kit (Sigma-Aldrich, CS0740) following the protocol of the product.

### Lysosomal acidification level assay

MCF7-shCtrl, shYAP and YAP Res cells were seeded in 6-well plates for 24 h and then detached by trypsin-EDTA. Cells were resuspended in 500 μL of fresh culture medium. We added 1 μL of LysoTracker Red DND 90 (50μM, Invitrogen, L7528) to each cell suspension and incubated it at 37°C for 10 min. The stained cells were assayed by flow cytometer (BD FACS AriaIII).

### Statistical analysis

Results are reported as the means±SEM. Comparisons between two groups were accomplished using an unpaired Student’s t test. *P*<0.05 was considered statistically significant.

## Results

### YAP knockdown sensitizes MCF7 cells to nutrient deprivation-induced apoptosis

**Fig 1:**
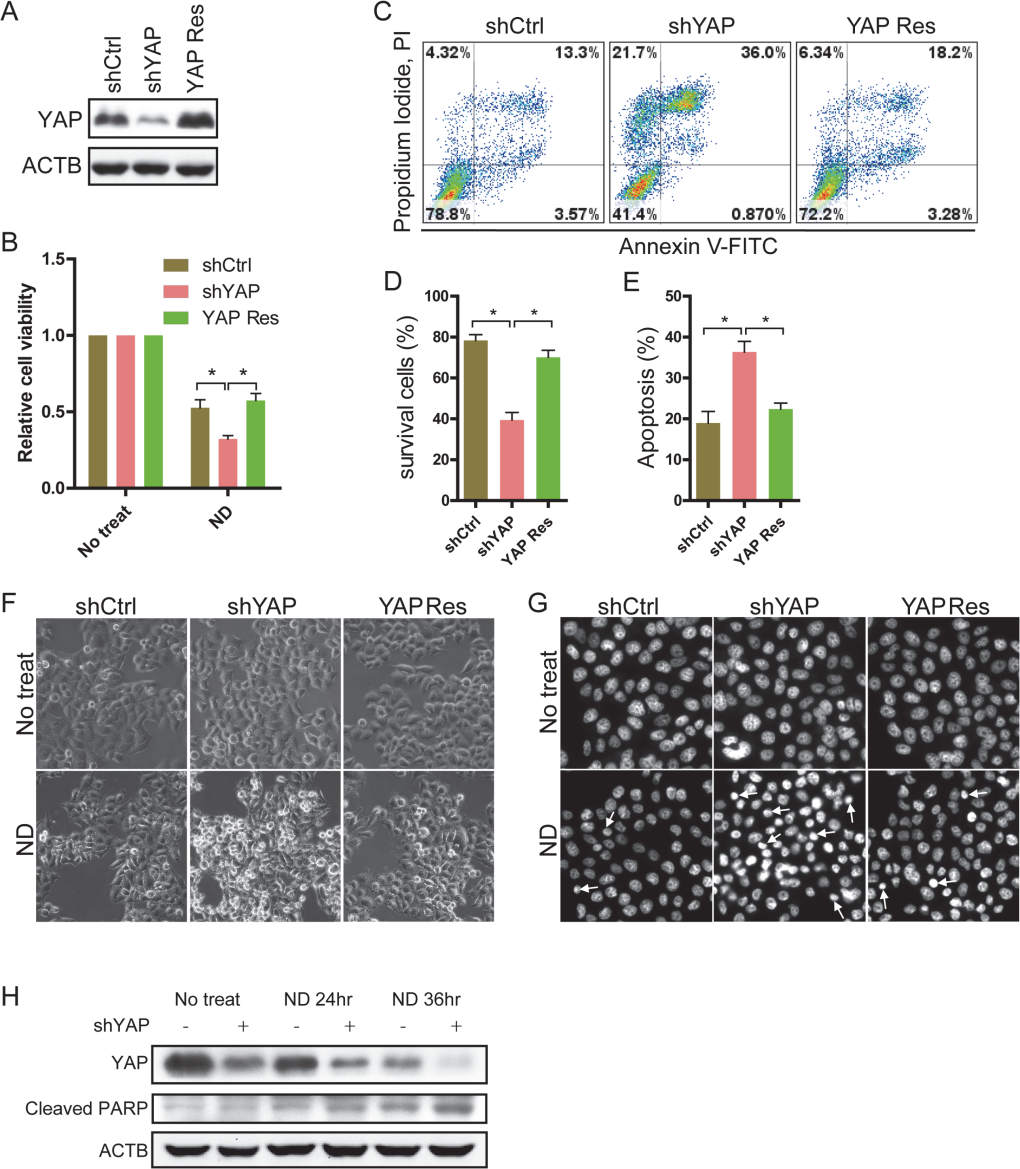
Nutrient deprivation-induced apoptosis in MCF7 cells is increased by YAP knockdown. **(A)** Expression of YAP in MCF7-shCtrl, shYAP and YAP Res cells was detected by Western blotting. **(B)** MCF7-shCtrl, shYAP and YAP Res cells were cultured in normal medium or EBSS for 36 h. Cell viability was determined by MTT assay. Data are shown as the means±SEM. *P<0.05, n = 3. **(C)** After 36 h of ND, cells were stained by Annexin V-FITC/PI and analyzed with flow cytometry. Representative results are shown. **(D)** Quantitative analysis of the percentage of surviving cells according to the results of (C) is shown. The numbers of surviving cells are expressed as the percentage of Annexin V-FITC/PI double negative cells out of the total number of cells in the gate. Data are shown as the means±SEM. *P<0.05, n = 3. **(E)** Quantitative analysis of the percentages of apoptotic cells, according to the results of (C) is shown. The numbers of apoptotic cells are expressed as the percentage of Annexin V-positive cells out of the total number of cells in the gate. Data are shown as the means±SEM. *P<0.05, n = 3. **(F)** Cells were maintained in normal medium or EBSS (ND) for 36 h. Cell morphology was examined by inverted phase contrast microscopy. Representative morphologic images are shown. **(G)** Nuclear morphology was investigated using Hoechst staining after treatment as in (F). **(H)** MCF7-shCtrl and shYAP cells were cultured in the indicated conditions for the indicated time. Total proteins were subjected to Western blotting for cleaved PARP. The results are representative of two independent experiments.

To determine if YAP protein levels interfere with the sensitivity of breast cancer cells to nutrient deprivation (ND), YAP was stably knocked down in MCF7 cells (control and YAP knockdown cell lines are called shCtrl and shYAP hereafter). YAP rescue protein was then stably expressed in MCF7-shYAP cells (hereafter called YAP Res) (Fig. 1A). These cells were cultured in EBSS to mimic the ND environment. After 36 h of ND, cell viability was determined by MTT assay. As shown in Fig. 1B, there were significantly more viable shCtrl cells compared with shYAP cells. Additionally, more viable cells were observed in YAP Res cells compared with shYAP cells. Consistent results were observed using MDA-MB-231 cells (S1 Fig.). These data indicate that YAP knockdown results in reduced survival under ND conditions. Next, we examined whether the reduced survival induced by YAP knockdown was due to apoptosis by performing flow cytometry using Annexin V-FITC/PI double staining. After ND treatment, there were more apoptotic and fewer survival populations in shYAP cells than in either shCtrl or YAP Res cells (Fig. 1C, 1D and 1E). There were also markedly more shrunken and detached shYAP cells than shCtrl or YAP Res cells (Fig. 1F). Further examination by Hoechst staining showed that apoptotic chromatin condensation was more frequently detected in shYAP cells under ND (Fig. 1G). The level of cleaved poly (ADP-ribose) polymerase (PARP), a marker of cells undergoing apoptosis [33], increased markedly in shYAP cells compared to shCtrl cells after ND treatment (Fig. 1H). In line with a recent study by Liang et al [34], we observed that after ND treatment, YAP levels decreased with time, which possibly increased the apoptosis. Altogether, these data indicate that YAP may play an important role in protecting MCF7 cells from apoptosis in response to ND stress.

### Autophagy is crucial for YAP to protect MCF7 cells from apoptosis under ND conditions

**Fig 2:**
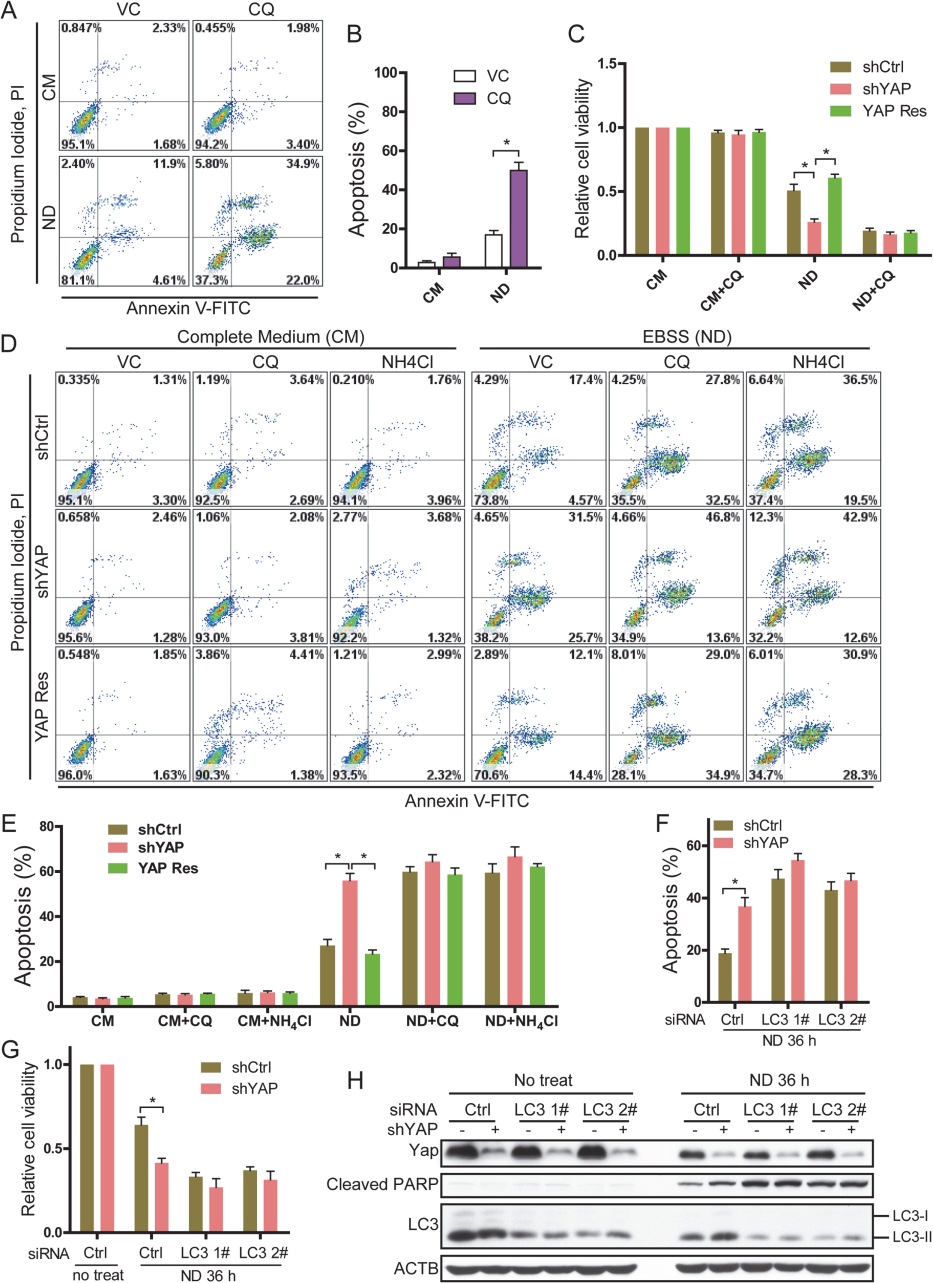
The different sensitivity to nutrient deprivation (ND) caused by YAP is attenuated after autophagy inhibition. **(A)** MCF7 cells were cultured under the indicated conditions for 36 h without chloroquine (CQ) or for 18 h with CQ, stained by Annexin V-FITC/PI, and analyzed with flow cytometry. Representative results are shown. **(B)** Quantitative analysis of percentages of apoptotic cells according to results of (A) are expressed as the percentage of Annexin V-positive cells out of the total number of cells in the gate. Data are shown as the means±SEM. *P<0.05, n = 3. **(C)** After 38 h (without CQ) or 18 h (with CQ) of indicated treatments, MCF7-shCtrl, shYAP and YAP Res cell viability was determined by MTT assay. *P<0.05, n = 3. **(D)** After 38 h (without CQ/NH_4_Cl) or 18 h (with CQ/NH_4_Cl) of indicated treatments, MCF7-shCtrl, shYAP and YAP Res cells were stained by Annexin V-FITC/PI and analyzed with flow cytometry. **(E)** Quantitative analysis of percentages of apoptotic cells according to the results of (D) was assayed as previously. Data are shown as the means±SEM. *P<0.05, n = 3. **(F)** MCF7-shCtrl and shYAP cells were transfected with control (Ctrl) and LC3 siRNA for 48 h and treated with ND for 36 h. Cells were then stained by Annexin V-FITC/PI and analyzed with flow cytometry. Quantitative analysis of percentages of apoptotic cells is derived from triplicates. *P<0.05, n = 3. **(G)** MCF7-shCtrl and shYAP cells were transfected with control (Ctrl) and LC3 siRNA for 48 h and cultured in normal medium or EBSS (ND) for 36 h. Cell viability was determined by MTT assay. *P<0.05, n = 3. **(H)** MCF7-shCtrl and shYAP cells were transfected with the indicated siRNA for 48 h and cultured in normal medium or EBSS (ND) for 36 h. Representative blots are shown. The experiment was repeated twice.

Nutrient deprivation (ND) induces the autophagy that promotes survival of different cancer cells [35]. We asked whether autophagy plays a role in the apoptotic increase in YAP knockdown MCF7 cells upon ND treatment. To test this, we first investigated whether autophagy was involved in MCF7 cell survival under ND conditions. Chloroquine (CQ) and NH_4_Cl suppress lysosomal degradation by increasing lysosomal pH, thus suppressing autophagy [18,35]. CQ and NH_4_Cl were therefore used to inhibit ND-induced autophagy in our study. MCF7 cells were cultured in normal medium and EBSS with or without CQ. As shown in Fig. 2A and 2B, 18 h of ND+CQ treatment significantly increased the number of apoptotic cells compared to ND-only conditions for 36 h. ND+CQ treatment for 36 h resulted in the breakdown of almost all cells (data not shown). CQ treatment alone for 18 h or 36 h barely interfered with cell apoptosis. This result suggests that normal autophagic flux is required for the survival of MCF7 cells under ND conditions. CQ can severely block autophagic flux by suppressing lysosomal degradation. When treated with ND + CQ, cell survival greatly decreased, and apoptosis occurred rapidly.

Next, we determined if the YAP knockdown decreased MC7 cell survival under ND conditions was mediated by autophagy. MTT assay showed that, when autophagy was inhibited, the viability of shCtrl, shYAP and YAP Res cells all decreased. Viability differences between these cell lines were no longer significant (Fig. 2C). Furthermore, Annexin V-FITC/PI staining showed that the apoptosis levels greatly increased in all three cell lines under ND+CQ and ND+NH_4_Cl conditions compared with ND alone. More importantly, the significant differences in the apoptotic levels between shYAP and shCtrl or YAP Res cells under ND conditions were attenuated following autophagy inhibition (Fig. 2D and 2E). Consistently, siRNA-mediated knockdown of LC3 in MCF7-shCtrl and shYAP cells attenuated the different levels of apoptosis (Fig. 2F), viability (Fig. 2G) and cleaved PARP (Fig. 2H) between MCF7-shCtrl and shYAP cells under ND conditions. These observations demonstrate that autophagy is critical for YAP to protect MCF7 cells from apoptosis under nutrient deprivation.

### YAP affects autophagic flux in breast cancer cells

**Fig 3:**
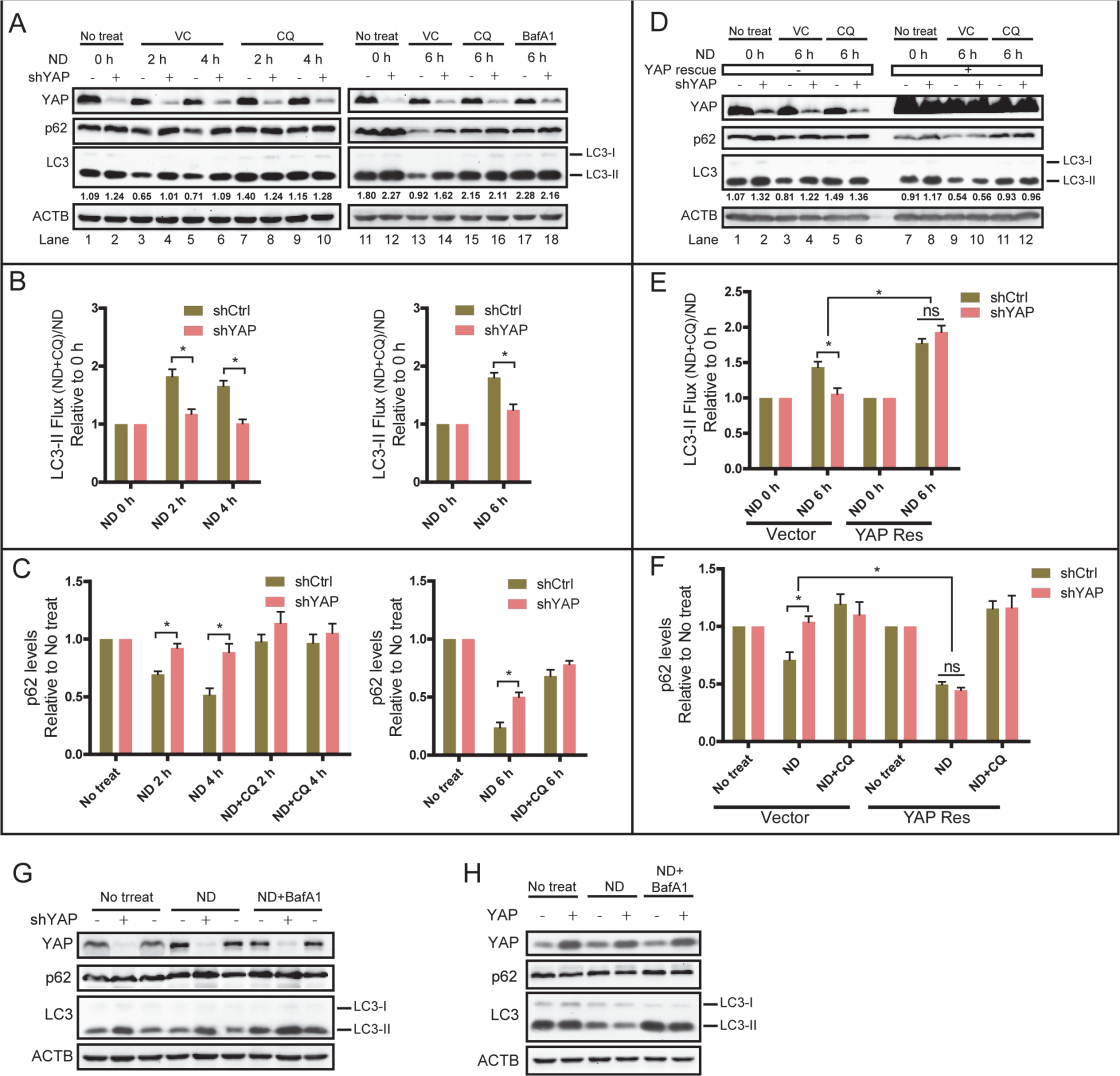
Knockdown of YAP inhibits autophagic flux in breast cancer cells. **(A)** MCF7-shCtrl and shYAP cells were cultured in normal medium (ND 0 h or no treatment) or maintained in EBSS (ND) +vehicle control (VC) or EBSS +CQ or Bafilomycin A1 (BafA1) for 2,4, and 6 h. Total proteins were subjected to Western blotting for the indicated antibodies. Representative results are shown. **(B)** Quantified relative LC3-II flux according to the Western blot results of (A) is expressed as the normalized LC3-II ratio calculated by the levels of LC3-II in the presence of CQ divided by the levels of LC3-II in absence of CQ. Data are derived from three independent experiments and shown as the means±SEM. *P<0.05. **(C)** Quantitative relative p62 levels according to results of (A) are expressed as p62 levels after different treatments divided by that of no treatment. Data are derived from three independent experiments and shown as the means±SEM. *P<0.05. **(D)** MCF7-shCtrl, shYAP and YAP-rescue-overexpression cells were cultured in normal medium (ND 0 h or no treatment) or maintained in EBSS (ND)+Vehicle Control (VC) or EBSS +CQ for 6 h. Total proteins were assayed by Western blotting. Representative results are shown. **(E)** Quantitative relative LC3-II flux according to Western blot results of (D) was assayed as previous. Data are derived from three independent experiments and shown as the means±SEM. *P<0.05. **(F)** Quantified relative p62 levels according to the results of (D) were assayed as previous. Data are derived from three independent experiments and shown as the means±SEM. *P<0.05. **(G)** MDA-MB-231-shCtrl and shYAP cells were cultured for 4 h in normal medium (no treatment), EBSS+VC (ND) or EBSS+BafA1. Total proteins were assayed by Western blotting with indicated antibodies. **(H)** MDA-MB231-Control and YAP overexpression cells were cultured for 6 h in normal medium (no treatment), EBSS+VC (ND) or EBSS+BafA1. Total proteins were assayed by Western blotting.

Next, we investigated whether aberrant YAP protein levels interfered with autophagy directly. Both LC3-II and p62 protein levels were used as indicators of autophagic flux. It has been reported that monitoring LC3-II levels in the presence and absence of inhibitors more accurately estimates lysosomal degradation during starvation-induced autophagy [18,36,37]. Thus, MCF7-shCtrl and shYAP cells were cultured under ND conditions in the presence or absence of lysosome inhibitors (CQ or Bafilomycin A1 (BafA1)) for different times. Whole-cell lysates were subjected to Western blotting for changes in LC3-II and p62. Previous studies showed that in MCF7 cells, EBSS treatment resulted in decreased LC3-II levels caused by faster degradation [38–40]. Consistently, in our experiment, ND treatment led to marked degradation of LC3-II, as well as p62, in MCF7-shCtrl cells at all time-points examined (Fig. 3A, lanes 3, 5,13), suggesting that ND increased the autophagic flux in MCF7-shCtrl cells. However, there was less LC3-II and p62 degradation in shYAP cells upon ND treatment than in shCtrl cells (Fig. 3A, lanes 4, 6, 14). If treated with ND+CQ or ND+BafA1, LC3-II and p62 degradation in both shCtrl and shYAP cells was inhibited (Fig. 3A, lanes 7–10, 15–18). Quantified relative LC3-II flux and p62 level analysis consistently showed that ND-induced autophagic flux was significantly inhibited by YAP knockdown (Fig. 3B and 3C). Expression of YAP rescue protein in shYAP cells eliminated the differences in autophagic flux between shCtrl and shYAP cells (Fig. 3D, lanes 3, 4 and 9, 10; Fig. 3E and 3F). Furthermore, increased LC3-II flux and p62 degradation were seen in cells expressing YAP rescue protein when cultured under ND stress (Fig. 3E and 3F). Knockdown of YAP in another breast cancer cell line, MDA-MB-231, also blocked LC3-II and p62 degradation (Fig. 3G). Consistently, over-expression of YAP in MDA-MB-231 increased LC3-II and p62 degradation (Fig. 3H). Immunofluorescence staining of endogenous LC3 puncta after ND treatment also obtained the consistent results (S2 Fig.). Together, these data demonstrate that YAP plays a role in increasing the autophagic flux of breast cancer cells under ND condition.

### YAP interferes with autophagic flux by enhancing autolysosome degradation

**Fig 4:**
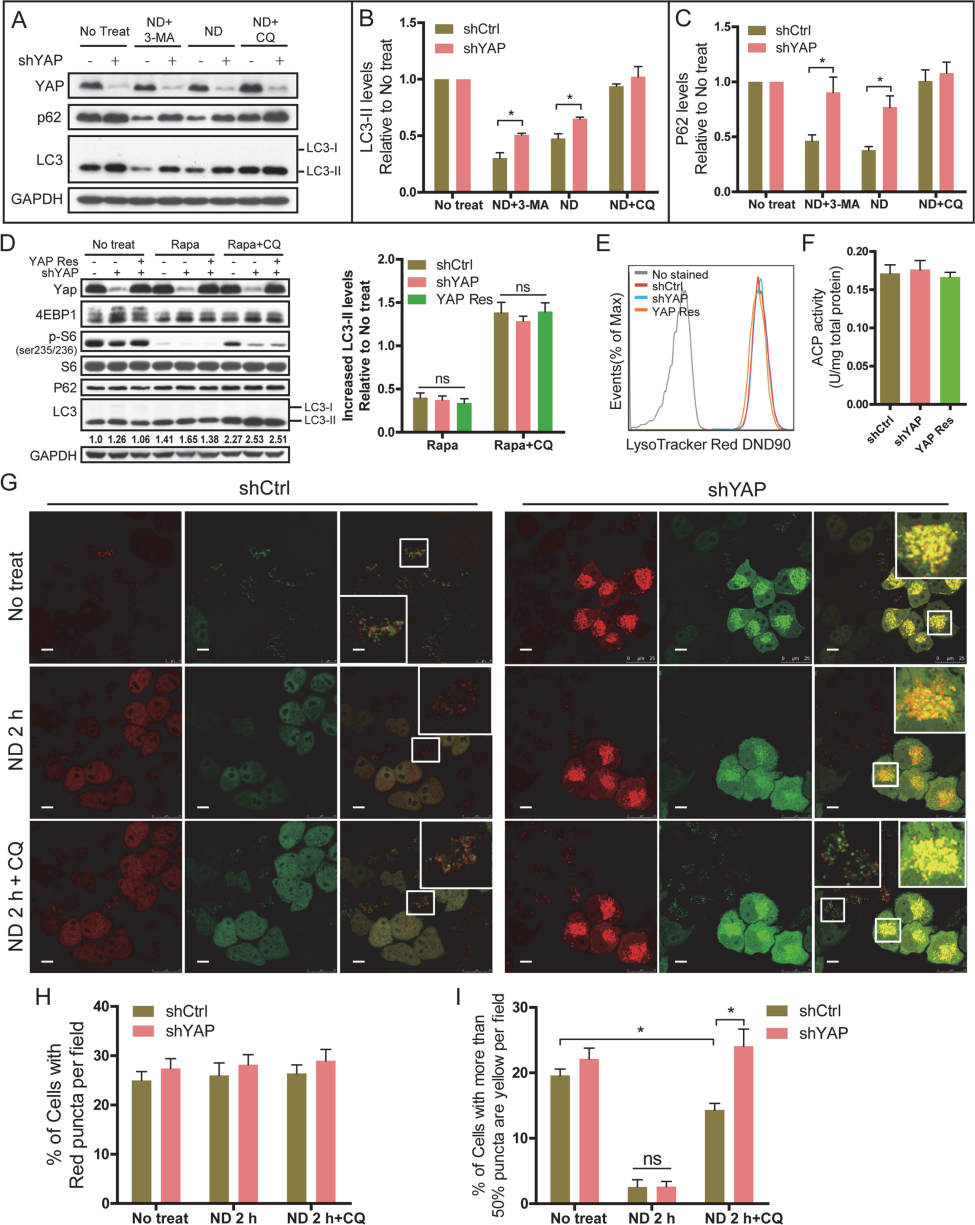
YAP interferes with autophagic flux via enhancing autolysosome degradation. **(A)** MCF7-shCtrl and shYAP cells were cultured for 6 h in normal medium (no treatment), EBSS (ND), EBSS+3-MA or EBSS+CQ. Total proteins were subjected to Western blotting. Representative results are shown. **(B)** Quantitative relative levels of LC3-II according to the results of (A) are expressed as LC3-II levels after different treatments (ND, ND+3-MA, ND+CQ) divided by that of no treatment. Data are derived from three independent experiments and shown as the means±SEM. *P<0.05. **(C)** Quantitative relative p62 levels according to the results of (A) are expressed as p62 levels after different treatments divided by that of no treatment. Data are derived from three independent experiments and shown as the means±SEM. *P<0.05. **(D)** MCF7-shCtrl, shYAP and YAP Res cells were treated with rapamycin (Rapa) or Rapa +CQ for 4 h. Total proteins were subjected to western blotting. Increased LC3-II levels were quantified as LC3-II levels after different Treats (Rapa, Rapa+CQ) subtract that of no treat. Quantitative data are derived from three independent experiments and shown as the means±SEM. *P<0.05. **(E)** Lysosomal acidification levels of MCF7-shCtrl, shYAP and YAP Res cells were assayed by flow cytometry after staining with LysoTracker Red. **(F)** ACP activities of MCF7-shCtrl, shYAP and YAP Res cells were assayed. **(G)** MCF7-shCtrl-tfLC3 and MCF7-shYAP-tfLC3 cells were starved in EBSS for 2 h. CQ was then added to elevate the lysosomal pH. Images of live cells were taken with a confocal microscope (Scale bar: 10 μm). **(H)** Percentages of cells with red puncta per field were counted and calculated. Data are shown as the means±SEM. *P<0.05, n = 3. **(I)** Percentages of cells with more than half the puncta yellow per field were counted and calculated. Data are shown as the means±SEM. *P<0.05, n = 3.

Autophagy is a highly dynamic, multi-step process that can be modulated at several steps, including autophagosome formation, maturation, fusion with lysosomes to generate autolysosomes, and degradation [41]. To investigate which step was targeted by YAP, 3-methyladenine (3-MA) and CQ were used as inhibitors of autophagosome formation and degradation. We found that the differences in LC3-II and p62 degradation between MCF7-shCtrl and shYAP cells under ND were attenuated in the presence of CQ, but not 3-MA (Fig. 4A, 4B and 4C). Moreover, treatment of rapamycin (Rapa), an autophagosome formation inducer, did not lead to more increase of LC3-II level in shYAP cells compared with shCtrl and YAP Res cells (Fig. 4D). These data suggest that YAP might interfere with autophagy by targeting the degradation and not the formation step of autophagy.

Functional lysosomes are critical for the maturation and degradation steps of autophagy. Lysosomal acidification is necessary for the catalytic activity of lysosomal enzymes. Therefore, we next examined the lysosomal acidification level by flow cytometry after live shCtrl, shYAP and YAP Res cells were stained with LysoTracker. Our data showed that there were no significant differences in LysoTracker staining between the different cell lines (Fig. 4E). The activity of acid phosphatase (ACP), a representative lysosomal enzyme, was not affected by aberrant YAP protein levels (Fig. 4F). These data indicate that the effect of YAP on autophagy is not via changing the lysosomal acidification.

Tandem fluorescent mRFP-GFP-tagged LC3 (tfLC3) is designed to monitor autophagic flux [42]. The GFP signal is quenched in the acidic autolysosome, whereas mRFP is more stable. Therefore, colocalization of both GFP and mRFP fluorescence indicates an autophagosome that has not fused with a lysosome. In contrast, an mRFP signal without GFP corresponds to an autolysosome [43]. Cells cultured in EBSS led to increased quenching of the GFP signal, caused by more autophagosome/lysosome fusion and faster degradation of GFP-LC3 [44]. Chloroquine (CQ) and NH_4_Cl suppress lysosomal degradation by increasing lysosomal pH [18,35]. When treated with CQ or NH_4_Cl, the quenched GFP signal of tfLC3 localizing to autolysosomes will recover. In our experiment, MCF7-shCtrl and shYAP cells were stably transfected with a plasmid expressing tfLC3. The resulting cell lines were named MCF7-shCtrl-tfLC3 and MCF7-shYAP-tfLC3. When starved in EBSS for 2 h, the GFP signal was remarkably reduced in both cell lines, but the mRFP signal remained (Fig. 4G, 4H and 4I), suggesting that autophagosome/lysosome fusion occurred normally in both cell lines. However, when CQ was added to elevate lysosomal pH after 2 h of EBSS treatment, MCF7-shYAP-tfLC3 cells exhibited significantly more co-localization of GFP and mRFP fluorescence (yellow puncta) than MCF7-shYAP-tfLC3 cells (Fig. 4G, 4H and 4I). Similar results were obtained by using NH_4_Cl instead of CQ (data not shown). It has been reported that GFP-LC3 is degraded in a step-wise fashion in the autolysosome, in which free GFP fragments are first generated, but their accumulation depends on the capacity of lysosomal degradation [44]. The increased GFP signal in shYAP cells when CQ was added after 2 h of EBSS treatment indicated their reduced lysosomal degradation compared with shCtrl cells.

Together, these data indicate that YAP may increase autolysosome degradation, but does not interfere with the fusion of autophagosomes and lysosomes.

### YAP increase of autophagic flux is TEAD-dependent

**Fig 5:**
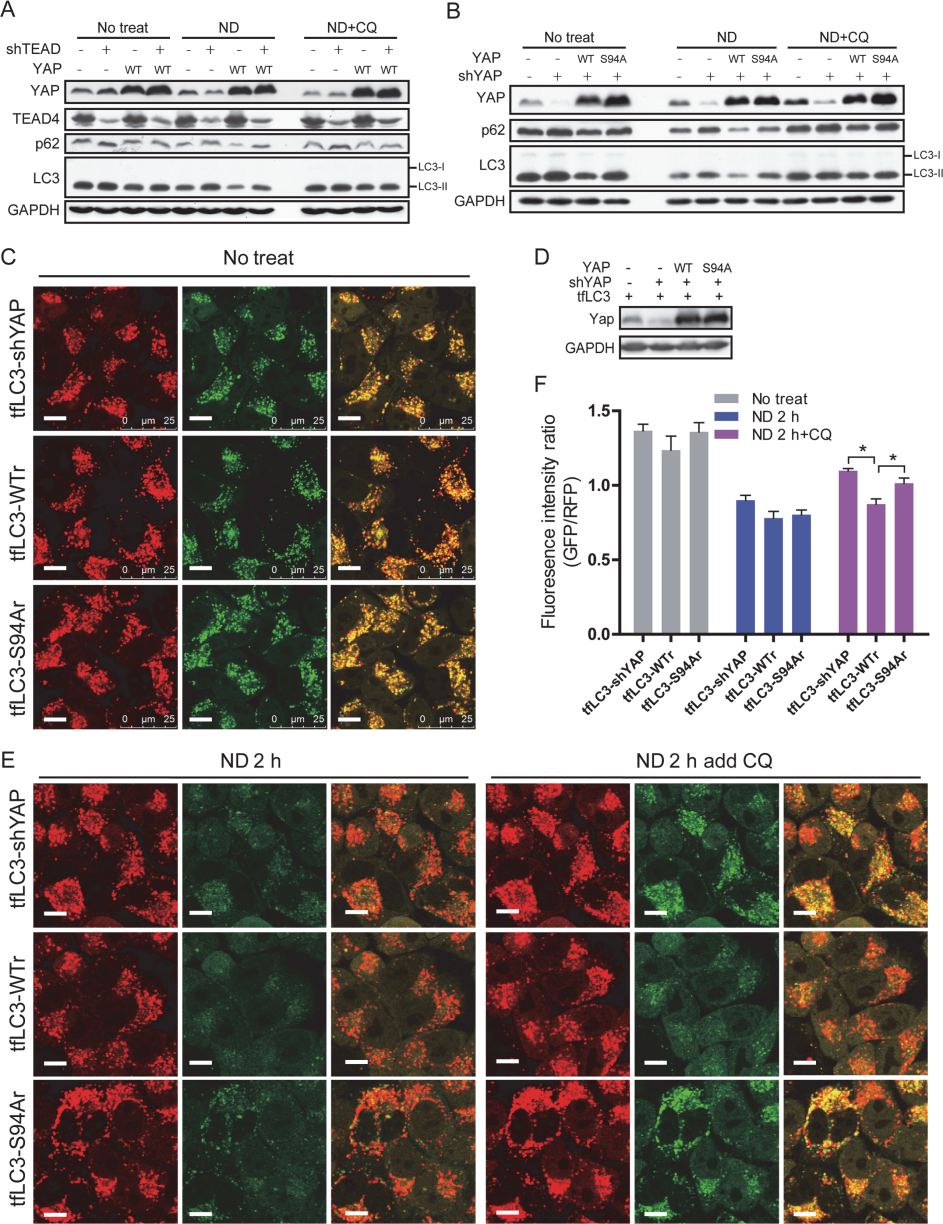
YAP-increased autolysosome degradation is TEAD-dependent. **(A)** TEAD1/3/4 was knocked down in normal and YAP-overexpressing MCF7 cells. Indicated cells were cultured for 6 h in normal medium (no treat), EBSS (ND) or EBSS +CQ. Total proteins were assayed by Western blotting. **(B)** MCF7-shYAP cells were stably infected with retrovirus expressing YAP-WT or YAP-S94A to generate WT Res and S94A Res cell lines. MCF7-shCtrl, shYAP, WT Ras and S94A Ras cells were cultured for 6 h in normal medium (no treatment), EBSS (ND) or EBSS +CQ. Total proteins were detected by Western blotting for indicated antibodies. **(C)** Images of normal cultured tfLC3-shYAP, tfLC3-WTr and tfLC3-S94Ar cells were taken with a confocal microscope. **(D)** YAP protein levels in MCF7-shCtrl-tfLC3, tfLC3-shYAP, tfLC3-WTr and tfLC3-S94Ar cell lines were determined. **(E)** tfLC3-shYAP, tfLC3-WTr and tfLC3-S94Ar cells were starved in EBSS for 2 h, and CQ was added to elevate lysosomal pH. Images of live cells were taken with a confocal microscope (Scale bar: 10 μm). **(F)** Average red and green fluorescent intensities per live cell in each well were valued by a high content cell assay microscope system. Green/red ratios were calculated and shown here. Data are shown as the means±SEM. *P<0.05, n = 3.

TEAD family transcription factors (TEADs) are essential for mediating YAP-dependent gene expression and are also required for YAP-induced cell functions [28]. To investigate whether TEADs are involved in the regulation of autophagy by YAP, TEAD1/3/4 was knocked down in normal and YAP-overexpressing MCF7 cells. Western blotting showed that ND-induced degradation of LC3-II and p62 was remarkably weakened in TEAD1/3/4 knockdown cells (Fig. 5A). In addition, re-expression of YAP-S94A, a YAP Ser94 to alanine (S94A) mutant that is defective in TEAD binding ability [28], did not enhance the ND-induced degradation of LC3-II and p62 as effectively as YAP-WT (Fig. 5B). Together, these data indicate the requirement of at least one of TEAD1/3/4 for YAP to increase autophagic flux.

To obtain further evidence that YAP increases autophagic flux via TEAD, cells with high-fluorescence-intensity puncta in MCF7-shYAP-tfLC3 cell line were selected by flow cytometry. High-fluorescence-intensity-puncta cells, called tfLC3-shYAP, were stably infected with retroviruses expressing YAP-WT or YAP-S94A to generate the tfLC3-WTr and tfLC3-S94Ar cell lines (Fig. 5C and 5D). After 2 h of starvation in EBSS, the GFP puncta signals in tfLC3-shYAP, tfLC3-WTr and tfLC3-S94Ar cell lines were quenched similarly (Fig. 5E and 5F). However, when CQ was added to elevate the lysosomal pH after 2 h of EBSS treatment, tfLC3-WTr cells exhibited significantly less colocalization of GFP and mRFP fluorescence than the other two cell lines (Fig. 5E and 5F). Taken together, these data indicate that YAP increases autolysosome degradation in a TEAD-dependent manner.

## Discussion

The role of YAP in breast cancer remains elusive. In this study, we found that YAP knockdown sensitizes breast cancer cells to nutrient deprivation-induced apoptosis. Our work further revealed that upon ND, YAP increases autolysosome degradation, thereby enhancing cellular autophagic flux to protect breast cancer cells from ND-induced apoptosis. Thus, our study highlights a role for YAP in promoting breast cancer cell survival upon ND stress.

Under conditions of metabolic stress such as ND, autophagy is induced to provide the nutrients and energy required for cell viability. For instance, autophagy may promote the survival of starved tumor cells in regions of the tumor with poor blood supply [45]. Accumulating evidence reveals that autophagy plays a critical role in breast cancer biology and therapy. A recent study demonstrated that Beclin 1 and autophagy are required for the tumorigenicity of breast cancer stem-like/progenitor cells [40]. Another study showed that the suppression of autophagy by the deletion of FIP200 (200 kDa FAK family interacting protein), an important regulator of autophagy in mammalian cells, inhibits mammary tumorigenesis [46]. Therefore, these data support a role for autophagy in breast cancer.

To date, the interplay between the Hippo/MST pathway and the cellular autophagy machinery has not been thoroughly investigated. Recently, Maejima et al. showed that the mammalian STE20-like kinase-1 (MST-1) subverts autophagy and promotes apoptosis in the heart [26], suggesting a role for the MST/YAP pathway in integrating autophagy and apoptosis during cellular stress. However, it is largely unknown whether autophagy plays a critical role in MST/YAP pathway-mediated tumor suppression. In the present work, we first provide evidence that YAP can protect MCF7 cells from nutrient deprivation-induced apoptosis, indicating a role for YAP in breast cancer survival under ND. Our data further reveal that this protective function of YAP is mediated by autophagy, consistent with a recent study by Liang et al reporting that nutrient starvation promotes YAP degradation concomitantly with the induction of autophagy in a chloroquine-sensitive manner [34]. Importantly, Liang et al revealed that mTOR regulates YAP degradation through autophagy [34]. More specifically, we revealed that YAP enhanced autophagosome degradation, thereby increasing the autophagic flux in nutrient deprived breast cancer cells. Moreover, TEADs are involved in the YAP-mediated regulation of autophagy. Thus, our findings suggest a novel mechanism, via targeting autophagy, by which YAP promotes breast cancer cell survival under ND. This notion is consistent with the recent discovery that MST phosphorylates the Thr108 residue in the BH3 domain of Beclin 1 to inhibit autophagy, thereby promoting apoptosis [26], as YAP’s biological function is generally inhibited by MST in the Hippo pathway. Although we show that YAP does not exert a direct effect on lysosome acidification and the fusion of autophagosomes with lysosomes in breast cancer cells under ND conditions, our data suggest that YAP and TEAD may regulate gene expression, leading to increased autophagy via enhancing autolysosome degradation. Thus, YAP and TEAD probably regulate the expression of lysosomal enzymes or proteins related to lysosomal enzyme sorting to the lysosome, such as M6P receptors. The detailed molecular mechanism underlying the YAP regulation of autophagy needs to be further investigated.

Given the growing amount of literature documenting both oncogenic and growth suppressive roles for YAP in breast cancer, the precise contribution of YAP to breast cancer may be complex. In this study, we show that in response to ND, YAP promotes breast cancer cell survival by augmenting autophagic flux. Therefore, our findings underscore YAP as a potential target for breast cancer treatment.

## Supporting Information

S1 Fig Overexpression of YAP enhances autophagic flux, but has no significant effect on ND-induced apoptosis in MCF7 and MDA-MB-231 cells. **(A)** MCF7-Ctrl and YAP cells were cultured in normal medium (ND 0 h or no treatment) or maintained in EBSS (ND) +vehicle control (VC) or EBSS +CQ for 6 h. Total proteins were subjected to Western blotting for indicated antibodies. Representative results are shown. **(B)** Quantified relative LC3-II flux according to Western blot results of (A). Data are derived from three independent experiments and shown as the means±SEM. *P<0.05. **(C)** Quantitative relative p62 levels according to the results of (A). *P<0.05. **(D)** MCF7-Ctrl and YAP cells were cultured in normal medium or EBSS (ND) for 36 h. Cell viability was determined by MTT assay. *P<0.05, n = 3. **(E)** After 36 h of ND, MCF7-Ctrl and YAP cells were stained by Annexin V-FITC/PI and analyzed with flow cytometry. Representative results are shown. Quantitative analysis of percentages of apoptotic cells is derived from triplicates. *P<0.05. **(F)** After 18 h (without CQ) or 8 h (with CQ) of indicated treatments, MDA-MB-231-shCtrl and shYAP cell viability was determined by MTT assay. *P<0.05, n = 3. **(F)** After 18 h of ND, MDA-MB231-Ctrl and YAP cell viability was determined by MTT assay. *P<0.05, n = 3.
(TIF)

S2 Fig Knockdown of YAP results in more LC3 puncta maintained in breast cancer cells under ND conditions. **(A)** Immunofluorescent images of LC3 in MCF7-shCtrl, shYAP and YAP Res cells, after treatment with ND or ND+CQ condition for 2 h. Representative images are shown. Quantification of LC3 puncta was determined from three independent experiments in which at least 30 cells with more than 10 puncta were counted. *P<0.05. **(B)** Immunofluorescent images of LC3 in MDA-MB-231-shCtrl and shYAP cells, after treatment with ND or ND+CQ for 2 h. Representative images are shown. Quantification of LC3 puncta was determined likewise.
(TIF)
