# Supplementary figures and images for: YAP Enhances Autophagic Flux to Promote Breast Cancer Cell Survival in Response to Nutrient Deprivation
Source: PLoS One. 2015 Mar 26;10(3):e0120790. doi: 10.1371/journal.pone.0120790 (PMC4374846; doi:10.1371/journal.pone.0120790)

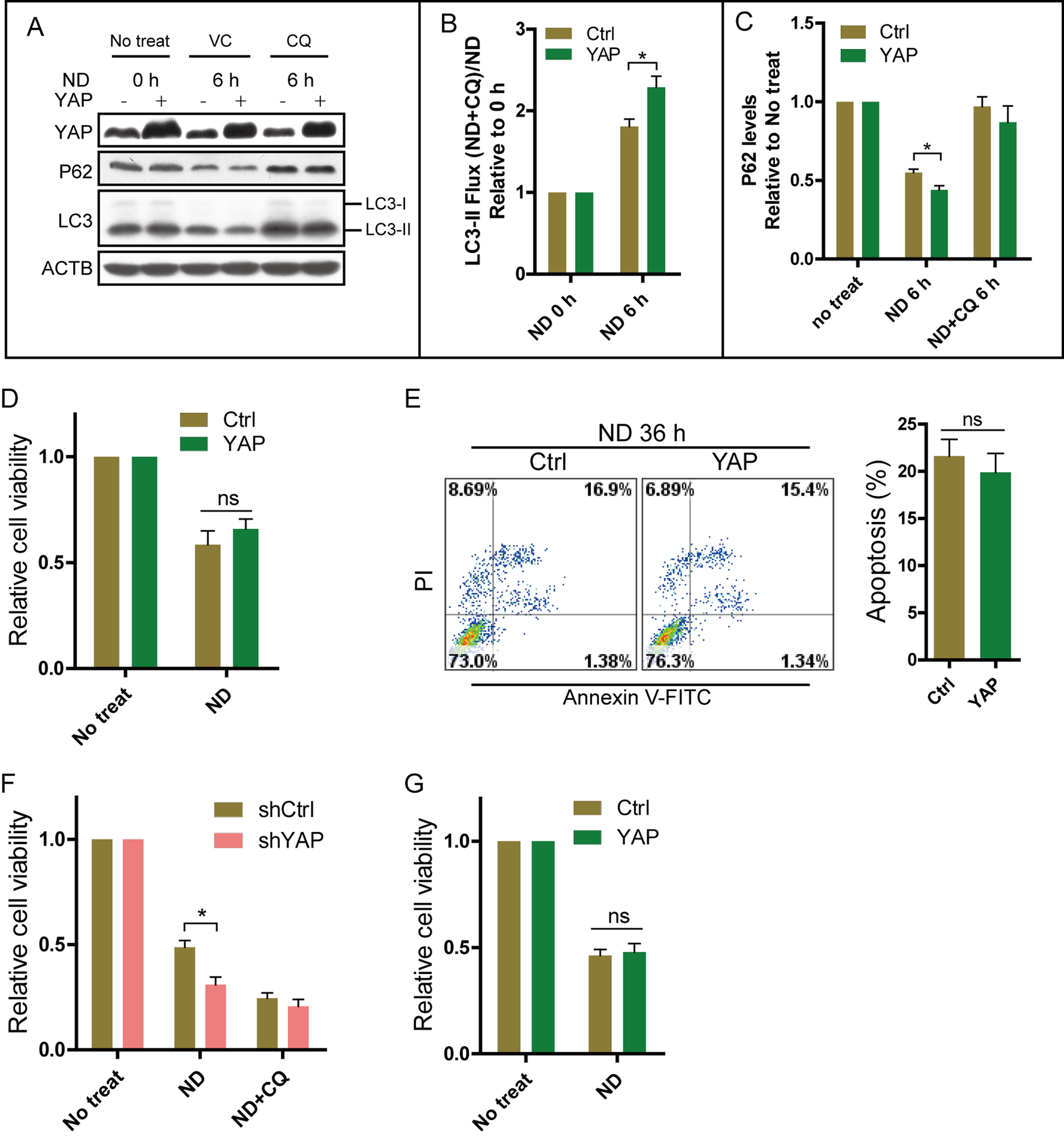

Supplement: S1 Fig — (A) MCF7-Ctrl and YAP cells were cultured in normal medium (ND 0 h or no treatment) or maintained in EBSS (ND) +vehicle control (VC) or EBSS +CQ for 6 h. Total proteins were subjected to Western blotting for indicated antibodies. Representative results are shown. (B) Quantified relative LC3-II flux according to Western blot results of (A). Data are derived from three independent experiments and shown as the means±SEM. *P<0.05. (C) Quantitative relative p62 levels according to the results of (A). *P<0.05. (D) MCF7-Ctrl and YAP cells were cultured in normal medium or EBSS (ND) for 36 h. Cell viability was determined by MTT assay. *P<0.05, n = 3. (E) After 36 h of ND, MCF7-Ctrl and YAP cells were stained by Annexin V-FITC/PI and analyzed with flow cytometry. Representative results are shown. Quantitative analysis of percentages of apoptotic cells is derived from triplicates. *P<0.05. (F) After 18 h (without CQ) or 8 h (with CQ) of indicated treatments, MDA-MB-231-shCtrl and shYAP cell viability was determined by MTT assay. *P<0.05, n = 3. (F) After 18 h of ND, MDA-MB231-Ctrl and YAP cell viability was determined by MTT assay. *P<0.05, n = 3. (TIF) [file pone.0120790.s001.tif]

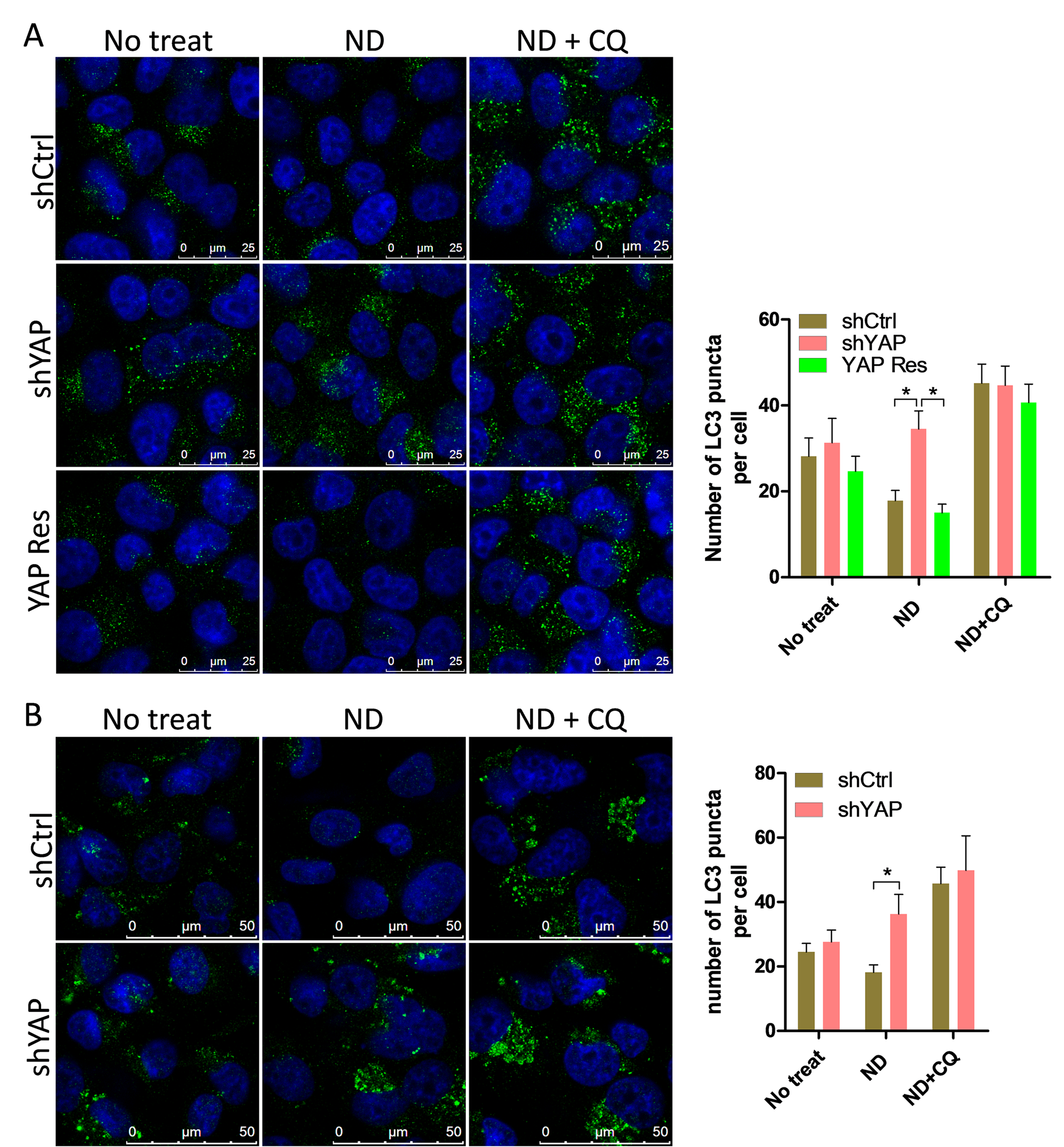

Supplement: S2 Fig — (A) Immunofluorescent images of LC3 in MCF7-shCtrl, shYAP and YAP Res cells, after treatment with ND or ND+CQ condition for 2 h. Representative images are shown. Quantification of LC3 puncta was determined from three independent experiments in which at least 30 cells with more than 10 puncta were counted. *P<0.05. (B) Immunofluorescent images of LC3 in MDA-MB-231-shCtrl and shYAP cells, after treatment with ND or ND+CQ for 2 h. Representative images are shown. Quantification of LC3 puncta was determined likewise. (TIF) [file pone.0120790.s002.tif]
